# Analysis of chromatin accessibility in peripheral blood mononuclear cells from patients with early-stage breast cancer

**DOI:** 10.3389/fphar.2024.1465586

**Published:** 2024-09-23

**Authors:** Longjie Xia, Jiamin Lu, Yixuan Qin, Runchun Huang, Fanbiao Kong, Yu Deng

**Affiliations:** ^1^ Department of Cosmetology and Plastic Surgery Center, The People’s Hospital of Guangxi Zhuang Autonomous Region, Guangxi Academy of Medical Sciences, Nanning, China; ^2^ Department of General Surgery, Guangzhou First People’s Hospital, Guangzhou, China; ^3^ Department of Plastic Surgery, The First Affiliated Hospital of Sun Yat-Sen University, Guangzhou, China; ^4^ Department of Colorectal and Anal Surgery, The People’s Hospital of Guangxi Zhuang Autonomous Region, Guangxi Academy of Medical Sciences, Nanning, China

**Keywords:** breast cancer, peripheral blood mononuclear cells, chromatin transposase sequencing, transcription factor, ATAC-seq

## Abstract

**Objective:** This study was aimed at exploring a specific open region of chromatin in the peripheral blood mononuclear cells (PBMCs) of patients with breast cancer and evaluating its feasibility as a biomarker for diagnosing and predicting breast cancer prognosis.

**Methods:** We obtained PBMCs from breast cancer patients and healthy people for the assay for transposase-accessible chromatin (ATAC) sequencing (n = 3) and obtained the GSE27562 chip sequencing data for secondary analyses. Through bioinformatics analysis, we mined the pattern changes for chromatin accessibility in the PBMCs of breast cancer patients.

**Results:** A total of 1,906 differentially accessible regions (DARs) and 1,632 differentially expressed genes (DEGs) were identified via ATAC sequencing. The upregulated DEGs in the disease group were mainly distributed in the cells, organelles, and cell-intima-related structures and were mainly responsible for biological functions such as cell nitrogen complex metabolism, macromolecular metabolism, and cell communication, in addition to functions such as nucleic acid binding, enzyme binding, hydrolase reaction, and transferase activity. Combined with microarray data analysis, the following set of nine DEGs showed intersection between the ATAC and microarray data: JUN, MSL2, CDC42, TRIB1, SERTAD3, RAB14, RHOB, RAB40B, and PRKDC. HOMER predicted and identified five transcription factors that could potentially bind to these peak sites, namely NFY, Sp 2, GFY, NRF, and ELK 1.

**Conclusion:** Chromatin accessibility analysis of the PBMCs from patients with early-stage breast cancer underscores its potential as a significant avenue for biomarker discovery in breast cancer diagnostics and treatment. By screening the transcription factors and DEGs related to breast cancer, this study provides a comprehensive theoretical foundation that is expected to guide future clinical applications and therapeutic developments.

## 1 Introduction

Breast cancer is the first among the major malignancies that threaten the lives of female patients. Early diagnosis and treatment are key to improving the prognosis of breast cancer, so an increasing number of tumor predictive markers are being widely studied and applied in clinical practice.

The detection of peripheral blood mononuclear cells (PBMCs) and chromatin transposase sequencing such as the high-throughput assay for transposase-accessible chromatin sequencing (ATAC-seq) can provide more sensitive and specific guidance in the diagnosis and treatment of cancer patients (Ding et al., 2020). PBMC testing can be used to detect and analyze circulating tumor cells (CTCs), which are highly relevant to breast-cancer-metastasis-related studies. CTCs are the means by which tumor cells spread to other parts of the body through the blood or lymphatic fluid and constitute one of the important links in breast cancer metastasis. CTC testing can help physicians and researchers detect metastasis risks early, thereby guiding individualized treatment; it can also be used to explore the heterogeneity of gene expressions between individual tumor cells, providing insights into the molecular mechanisms of tumor development (Ding et al., 2020). ATAC-seq technology can be combined with other methods, such as RNA chip data and ChIP-seq, to further explore the mechanisms of initiation and development of breast cancer (Wang et al., 2021).

In recent years, given the rapid development of multiple omics, researchers have attempted to understand the mechanisms of various organisms. Therefore, we also adopted the multi-omics method combined with ATAC-seq and RNA chip data to explore the gene expressions of PBMCs; we also investigated the relationships between chromatin accessibility from the level of transcriptomics and epigenetic omics to explore the molecular mechanism and genetic bases of early-stage breast cancer to enable prediction of the potential therapeutic targets of breast cancer.

## 2 Materials and methods

### 2.1 Acquisition of the specimens

The blood samples required for the study were obtained through the Breast Surgery Department of Guangzhou First People’s Hospital from three early-stage breast cancer patients and three healthy adult volunteers. This study was approved by the Ethics Committee of Guangzhou First People’s Hospital (approval no. K-2023-019-01). All clinical studies were conducted in accordance with the principles of the Declaration of Helsinki.

### 2.2 Acquisition, processing, and purification of PBMC specimens

Three women with early-stage breast cancer were selected as the experimental group, while three women without breast diseases were chosen as the control group. Blood samples were extracted from these subjects from the forearm; we obtained 5 mL of whole blood from each subject, which was placed in appropriate tubes (BD Vacutainer™) containing ethylenediamine tetraacetic acid, mixed for 8–10 times, and marked with the patient name and outpatient/hospital number before being stored at 4°C and transported to the laboratory for cell treatment within 2 h. During processing the tubes were centrifuged for 30 min at 2,500 rpm using a centrifuge with a swing bucket rotor. The plasma layer was removed, and the remaining sample was poured into a 15-mL conical tube. Next, 5 mL of frozen phosphate-buffered saline (PBS) containing 2% fetal calf serum (FBS) was added to a separate tube, capped, and mixed in an inverted position. The contents were then poured into the 15-mL conical tube and centrifuged at 1,200 rpm for 10 min at room temperature; the supernatant was then discarded for ATAC detection.

### 2.3 ATAC sequencing

The sample used for sequencing contained approximately 5 × 10^4^ cells in 100–200 μL, and the cell survival was controlled above 90% as much as possible. Then, 1 M of DNase was added in the ratio of 1:50 and mixed at 37°C for 30 min; this sample was centrifuged at 500*g* for 5 min, and the supernatant was carefully discarded. Next, 1 mL of precooled EPITM ATAC lysis buffer was added to the sample and mixed in an ice bath for 3 min before being centrifuged at 500*g* for 10 min; during centrifugation, the 50 µL transposase reaction system was configured with 35 μL of ddH_2_O, 10 μL of 5 × TT buffer, and 5 µL of Tn5 mix. The supernatant was then removed, and the nuclei were collected and added to the reaction system before mixing thoroughly 20 times. Following this, the samples were incubated for 30 min at 37°C and agitation at 1,000 rpm; lastly, the DNA was extracted from the incubated samples.

The raw data were obtained in the fastq format using fastp software (https://github.com/OpenGene/fastp); this procedure controls the raw data, including IP samples and input samples, and performs adaptor removal, repetitive sequence, and low quality sequences to yield clean data in the fastq format. Then, FastQC (https://github.com/s-andrews/FastQC) was applied to this clean data for quality control analysis. The clean reads data were then aligned with the reference genome using BWA software (version 0.7.17-r1188).

The data were further processed after comparing the bam files. The mitochondrial genome and duplicates were removed, where the duplicate refers to the sequence of reads to the genome at exactly the base and alignment with the reference genome. To avoid the impacts of these replications on subsequent analyses, we used Picard to remove the duplicates. Next, we used bedtools to remove the blacklist region. For reads on the positive strand, the starting position of alignment was +4, and for reads on the negative strand that are 5 bp to the left, the starting position of the alignment was -5 bp. We used the deeptools-alignmentSieve software (version: 3.5.1) to remove the offset reads, and HOMER was used to predict the motif sequences in the possible peak binding data.

### 2.4 GSE27562 chip data download and standardization

We downloaded the GSE27562 dataset from the NCBI gene expression omnibus (GEO) database (http://www.ncbi.nlm.nih.gov/geo) to obtain the chip data. This dataset mainly includes information from female patients diagnosed with breast cancer, patients with benign breast masses, patients with negative molybdenum targets, and patients after breast cancer surgery. We extracted the data of 57 female patients diagnosed with breast cancer and 31 patients without abnormalities as the control groups, including their Affymetrix cel and probe annotation files for the subsequent analyses. The platform used for the chip data is the GPL570 [HG-U133_Plus_2] Affymetrix Human Genome U133 Plus 2.0 Array (Affymetrix Company, United States).

After successfully downloading the data from BRAINARRAY and the GeneChip custom chip description file (CDF) from GENCODE, the data were background corrected and normalized using Affymetrix power tools software. Then, the gene-level probe set was mapped to the human GENCODE annotation (version 28) using a custom perl script. Only the RNA in the GENCODE database with probe-set annotation was retained as “PROTEIN-CODING,” while the other genes were filtered out. The rationality of line data normalization in the boxplot was assessed with log2PM. The differentially expressed genes (DEGs) were defined as genes with |log2FC| > 0.5 and adjusted *p* < 0.05. DEGs from the breast cancer and normal populations from the ATAC-seq and microarray reanalysis were retrieved for intersection analysis using the Venn diagram.

### 2.5 Data normalization and batch effect correction

To ensure comparability and reliability of our data analyses, we implemented robust normalization and batch effect correction. For the ATAC-seq data, we used the fragments per kilobase of transcripts per million mapped reads (FPKM) method to normalize the sequencing depth across samples, which mitigated the impacts of varying sequencing depths. The GSE27562 microarray data were processed for background correction, normalization, and probe-level signal summarization using the robust multiarray average (RMA) method. To address potential batch effects, we applied the “ComBat” method to the ATAC-seq data and used the “sva” R package for the GSE27562 data. These procedures effectively reduced the technical variability and enhanced the consistency and accuracy of the downstream analyses.

## 3 Results

### 3.1 Baseline and ATAC data quality inspections

We selected three women with early-stage breast cancer as the experimental group and three healthy adult women as the control group. The experimental group did not receive any treatment for early breast cancer, while the women in the control group had no breast masses until presentation (Table 1).

**TABLE 1:** Clinical patient information.

Group	Sample number	Age	Sex	Diagnosis	Surgical operation	Pathological type	Clinical stage
Experimental group	CSW	42	Female	Breast cancer	Denied	HR-/HER2+	cTisN0M0
	CXH	52	Female	Breast cancer	Denied	HR-/HER2-	cT1N0M0
	QXL	63	Female	Breast cancer	Denied	HR+/HER2-	cT2N0M0
Control group	CYZ	64	Female	Normal	Denied	None	0
	S13	48	Female	Normal	Denied	None	0
	S8	48	Female	Normal	Denied	None	0

The ATAC-seq quality control results are presented in Table 2, for which we observed the accessible regions and found that all specimens had 99% match with the genome (Table 3).

**TABLE 2:** ATAC quality controlled results.

Sample name	Number of original sequences	Total base numbers	Total number of sequences controlled and paired	Total number of bases that are quality-controlled and paired	GC ratio
CSW	118,518,974	1.78e+10	94,544,598	1.12e+10	0.445
CXH	146,227,086	2.19e+10	113,165,584	1.31e+10	0.443
CYZ	191,688,422	2.88e+10	140,223,270	1.58e+10	0.45
QXL	122,565,338	1.84e+10	89,271,816	8.85e+09	0.451
S13	218,332,778	3.27e+10	176,448,968	2.27e+10	0.437
S8	238,892,846	3.58e+10	198,617,718	2.53e+10	0.443

**TABLE 3:** Analysis of the sequence alignment results.

Sample name	Total number of sequences	Number of sequences in the alignment	Comparison rate
CXH	113,165,584	113,056,514	99.9
QXL	89,271,816	89,195,666	99.91
CSW	94,544,598	94,466,193	99.92
S8	198,617,718	198,465,443	99.92
CYZ	140,223,270	140,107,764	99.92
S13	176,448,968	176,351,915	99.94

### 3.2 Analyses of association degree and accessible region data for breast cancer PBMC ATAC-seq samples

**FIGURE 1:**
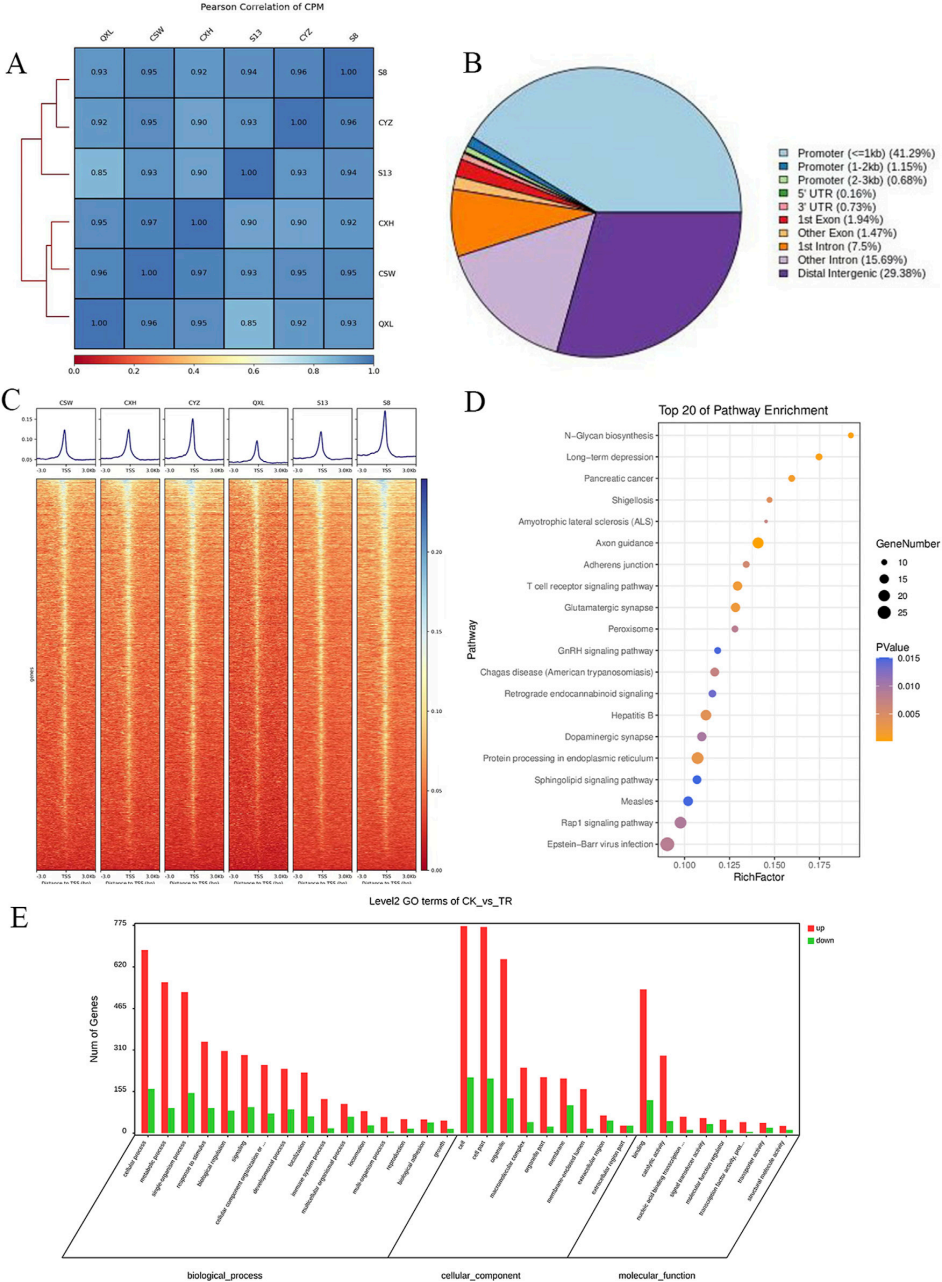
Analysis of the degree of correlation and accessible region data from ATAC-seq samples: **(A)** Pearson association analysis between the samples; **(B)** distribution of the accessible regions of the differential genes; **(C)** heatmap of the distance of the differentially accessible region (DAR) from the transcription start region (TSS) for each sample. **(D)** KEGG enrichment analysis of the top 20 pathways enriched by the differentially expressed genes (DEGs), where the dot sizes indicate the numbers of differential genes in each of the channels; the larger the dot size, the more are the number of genes. The colors indicate the *p*-values, where blue indicates *p* > 0.01, purple indicates *p* > 0.005 and *p* < 0.01, and yellow indicates *p* < 0.005. **(E)** Bar graph of the GO enrichment analysis of the DEGs, where red indicates that the DEG enriched subterms are upregulated in the disease group and green indicates the subterms that downregulate DEG enrichment in the disease group.

The correlations among the samples are shown in Figure 1A, and a total of 1,906 differentially accessible regions (DARs) and 1,632 DEGs were identified by ATAC-seq. From Figure 1B, it is seen that the DARs are mainly distributed in the promoter regions of the DEGs, followed by distal intergenic as well as other intronic regions. The ATAC-seq signals were enriched in the open chromatin regions and were positively correlated with the gene transcription activities. Heatmap analysis shows the enrichment distribution of the base sequence between the start positions (TSS) of the transcription factors (TFs) and the 3 kb upstream as well as downstream region of all genes: the signals of the two groups of cells are mostly located within ±3 kb. The overall trend of the control group is slightly higher than that of the experimental group. These results suggest intergroup differences, and the heatmaps of the distances between the DARs and transcription initiation regions of the samples are shown in Figure 1C.

Based on the Kyoto encyclopedia of genes and genomes (KEGG) and gene ontology (GO) enrichment analyses of the DEGs corresponding to the DARs, the differential genes were found to be enriched for N-glycan biosynthesis, T receptor signaling, peroxisome, GnRH signaling pathway, protein processing in the endoplasmic reticulum, and other pathways (Figure 1D). In the GO enrichment analysis, the DEGs of the experimental group were mainly distributed in the cells, organelles, and cell-membrane-related structures and were mainly responsible for biological functions like cell nitrogen complex metabolism, macromolecular metabolism, and cell communication, in addition to other functions like nucleic acid binding, enzyme binding, hydrolase enzyme reaction, and transferase activity (Figure 1E).

### 3.3 GEO online database for breast cancer PBMC microarray analysis

**FIGURE 2:**
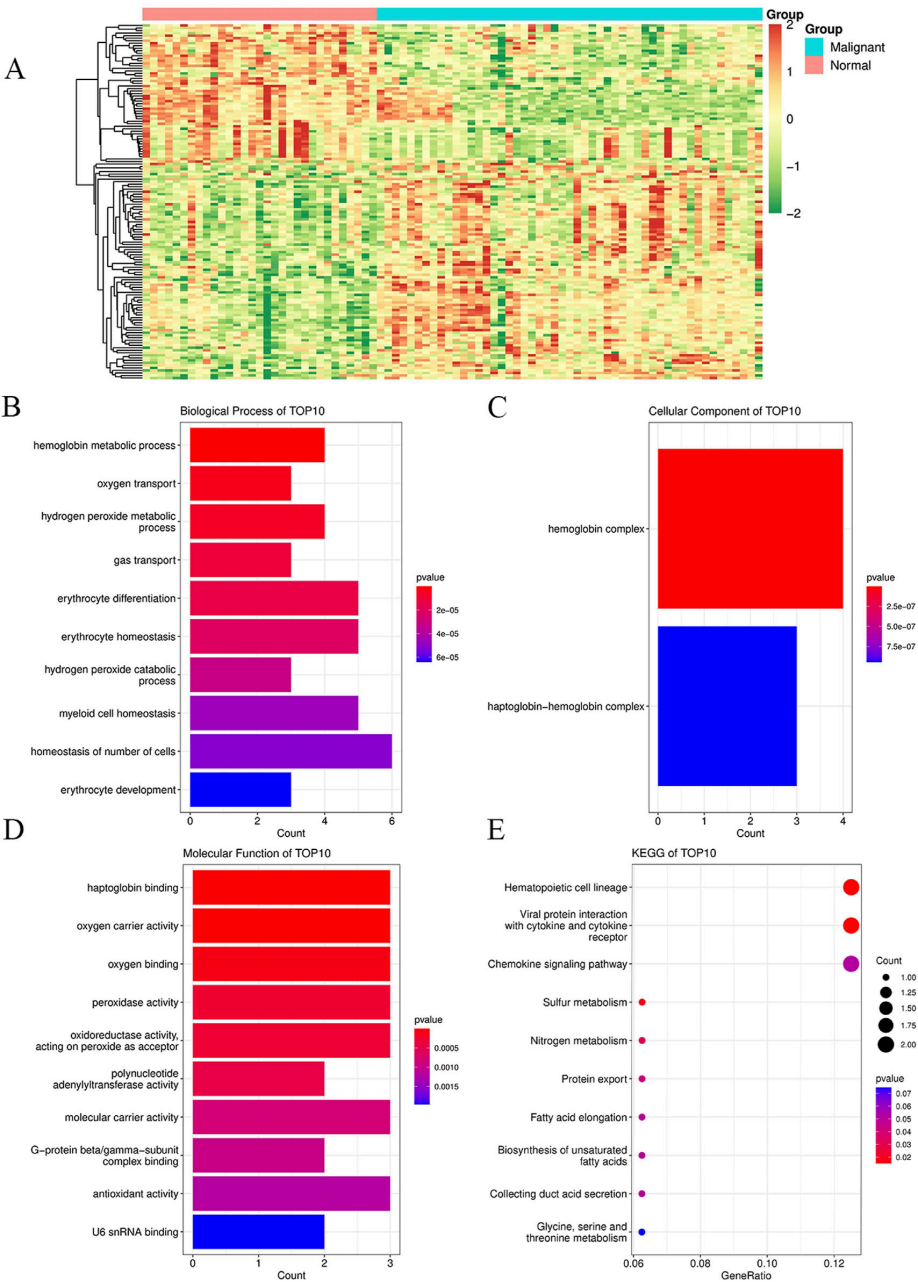
Enrichment analyses of DEGs and their functions in peripheral blood mononuclear cells between breast cancer patients and healthy controls for the GSE27562 dataset from the GEO database. **(A)** Heatmaps of all the genes in the GSE27562 dataset, where blue represents the tumor group and orange represents the normal group; the red data indicate increased expressions, green data indicate decreased expressions, and darker colors indicate higher gene expression value changes from the two extremes. **(B)** GO enrichment analysis of the DEGs for biological processes. **(C)** GO enrichment analysis of the DEGs for cellular components. **(D)** GO enrichment analysis of the DEGs for molecular functions. **(E)** KEGG enrichment analysis of the DEGs. In **(B–E)**, the dot sizes indicate the numbers of differential genes in each channel, with larger sizes implying more numbers and colors indicating the *p*-values.

We searched the GEO database for chip data related to the PBMCs of breast cancer and finally selected the GSE27562 dataset, which mainly includes information from female patients diagnosed with breast cancer, patients with benign breast masses, patients with negative molybdenum targets, and patients after breast cancer surgery. We extracted the population data for 57 female patients with breast cancers and 31 mammography cases for secondary analyses (Figure 2A). By setting |Log2FC| > 0.5 and *p* < 0.05 in these data, we found that 86 genes were upregulated and 55 genes were downregulated in the PBMCs of the experimental group. The GO and KEGG enrichment analyses of the DEGs revealed that the upregulated genes were primarily clustered in the GO hematopoiesis as well as hemoglobin-related subterms. The KEGG analysis showed that the upregulated genes were mainly enriched for MAPK signaling, TNF signaling, IL-17 post-absorption, GnRH signaling, and NOD-like receptor signaling (Figure 2E; Table 4), while the downregulated genes were mainly enriched for hematopoietic cell lines, cytokine receptors and their interactions with cellular proteins, sulfur metabolism, nitrogen metabolism, and protein outputs (Table 5).

**TABLE 4:** KEGG enrichment analysis of the top 10 upregulated differentially expressed genes in breast cancer patients and normal population.

ID	Description	Gene ratio	Q-value
hsa04010	MAPK signaling pathway	0.14	0.010452
hsa04668	TNF signaling pathway	0.12	0.001096
hsa05167	Kaposi-sarcoma-associated herpesvirus infection	0.12	0.00689
hsa05417	Lipid and atherosclerosis	0.12	0.008378
hsa05120	Epithelial cell signaling in *Helicobacter pylori* infection	0.10	0.001096
hsa04657	IL-17 signaling pathway	0.10	0.002538
hsa04933	AGE-RAGE signaling pathway in diabetic complications	0.10	0.002698
hsa04928	Parathyroid hormone synthesis, secretion, and action	0.10	0.002989
hsa04380	Osteoclast differentiation	0.10	0.006025
hsa04932	Non-alcoholic fatty liver disease	0.10	0.008378

**TABLE 5:** KEGG enrichment analysis of the top 10 downregulated differentially expressed genes in breast cancer patients and normal population.

ID	Description	Gene ratio	Q-value
hsa04640	Hematopoietic cell lineage	0.05	0.190316
hsa04061	Viral protein interactions with cytokines and cytokine receptors	0.05	0.190316
hsa04062	Chemokine signaling pathway	0.05	0.190316
hsa04060	Cytokine-to-cytokine-receptor interactions	0.05	0.257848
hsa04080	Neuroactive ligand–receptor interactions	0.05	0.282966
hsa00920	Sulfur metabolism	0.02	0.190316
hsa00910	Nitrogen metabolism	0.02	0.190316
hsa03060	Protein export	0.02	0.190316
hsa00062	Fatty-acid elongation	0.02	0.190316
hsa01040	Biosynthesis of unsaturated fatty acids	0.02	0.190316

Other functional analysis results are shown in Figures 2B–D.

### 3.4 Association analysis between ATAC-seq and gene microarray data

**FIGURE 3:**
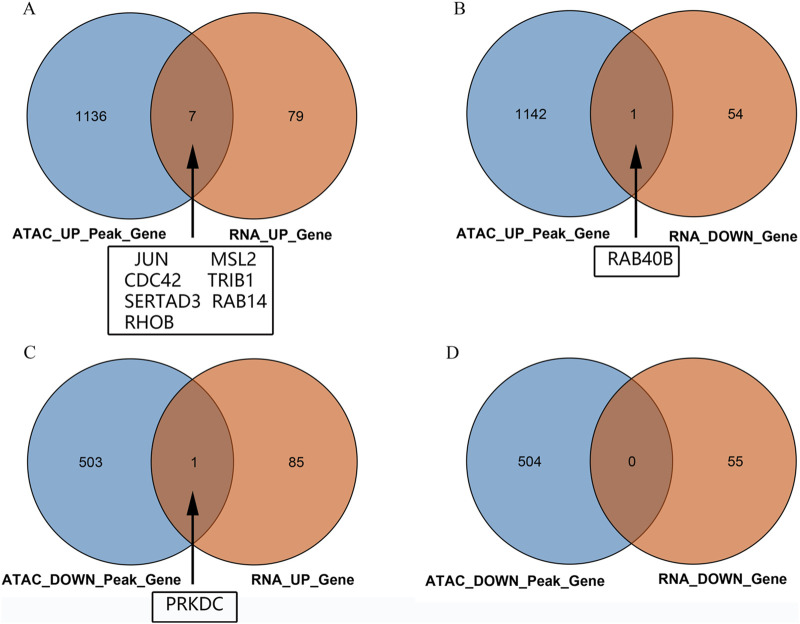
Venn diagrams of the intersections between the ATAC peaks of the peripheral blood mononuclear cells and chip data: **(A)** intersection of upregulated genes between ATAC and mRNA; **(B)** intersection of genes between upregulated ATAC and downregulated mRNA; **(C)** intersection of genes between downregulated ATAC and upregulated mRNA; **(D)** intersection of downregulated genes between ATAC and mRNA.

Intersection analysis of the peripheral blood ATAC sequencing and mRNA chip data from public databases revealed nine differentially expressed genes, namely JUN, MSL2, CDC42, TRIB1, SERTAD3, RAB14, RHOB, RAB40B, and PRKDC. Among these, seven DEGs were noted to be regulated by both mRNA data and ATAC sequencing, namely JUN, MSL2, CDC42, TRIB1, SERTAD3, RAB14, and RHOB (Figure 3A). The RAB40B gene showed ATAC upregulation and mRNA downregulation (Figure 3B), while the PRKDC gene showed ATAC downregulation and mRNA upregulation (Figure 3C); there were no intersecting genes between both downregulations (Figure 3D).

### 3.5 Motif predictions

The open regions of the chromatin may be bound by TFs to regulate gene expressions, and specific base sequences with high affinities to certain TFs are called as motifs. In the motif analysis, five specific TFs were identified: NFY, Sp 2, GFY, NRF, and ELK 1 (Table 6).

**TABLE 6:** HOMER predicts the top 20 transcription factors motifs with high binding probabilities in ATAC sequencing.

Rank	Motif/Name	Q-value	% of Targets Sequences with Motif	% of Background Sequences with Motif
1	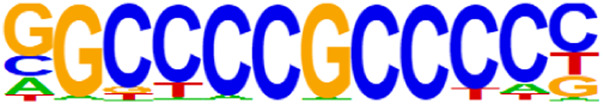 Sp1(Zf)/Promoter/Homer	<0.001	21.56%	7.24%
2	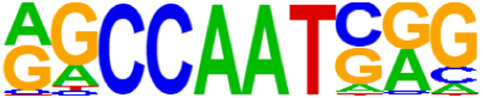 NFY(CCAAT)/Promoter/Homer	<0.001	21.20%	7.15%
3	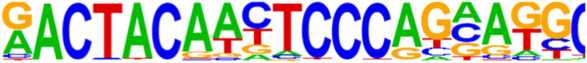 Ronin(THAP)/ES-Thap11-ChIP-Seq(GSE51522)/Homer	<0.001	4.93%	0.33%
4	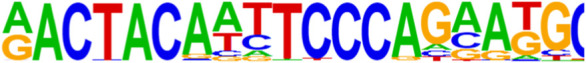 GFY-Staf(?,Zf)/Promoter/Homer	<0.001	5.35%	0.63%
5	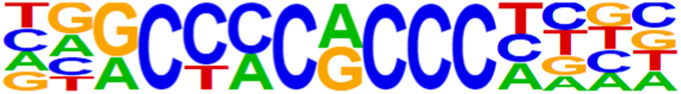 KLF3(Zf)/MEF-Klf3-ChIP-Seq(GSE44748)/Homer	<0.001	22.82%	10.29%
6	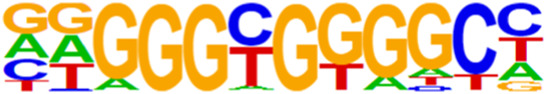 KLF1(Zf)/HUDEP2-KLF1-CutnRun(GSE136251)/Homer	<0.001	33.89%	18.61%
7	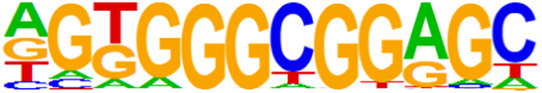 Sp5(Zf)/mES-Sp5.Flag-ChIP-Seq(GSE72989)/Homer	<0.001	35.10%	20.15%
8	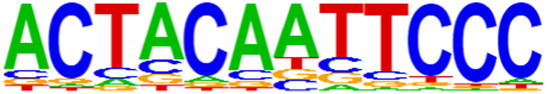 GFY(?)/Promoter/Homer	<0.001	4.93%	0.77%
9	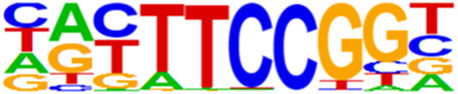 Elk4(ETS)/Hela-Elk4-ChIP-Seq(GSE31477)/Homer	<0.001	16.05%	7.18%
10	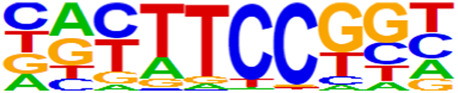 Fli1(ETS)/CD8-FLI-ChIP-Seq(GSE20898)/Homer	<0.001	24.34%	13.38%
11	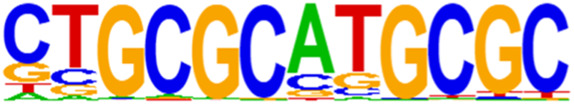 NRF1(NRF)/MCF7-NRF1-ChIP-Seq(Unpublished)/Homer	<0.001	9.50%	3.17%
12	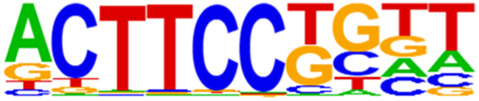 Elf4(ETS)/BMDM-Elf4-ChIP-Seq(GSE88699)/Homer	<0.001	20.83%	10.92%
13	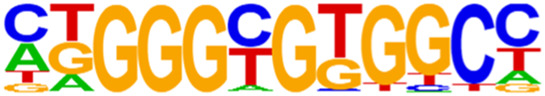 KLF6(Zf)/PDAC-KLF6-ChIP-Seq(GSE64557)/Homer	<0.001	32.90%	20.65%
14	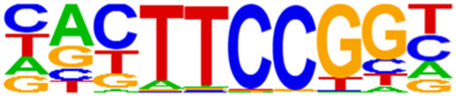 Elk1(ETS)/Hela-Elk1-ChIP-Seq(GSE31477)/Homer	<0.001	15.95%	7.52%
15	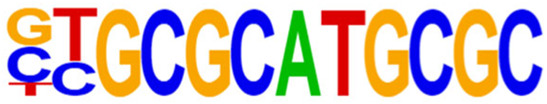 NRF(NRF)/Promoter/Homer	<0.001	9.86%	3.68%
16	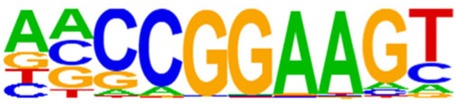 ELF1(ETS)/Jurkat-ELF1-ChIP-Seq(SRA014231)/Homer	<0.001	14.74%	6.87%
17	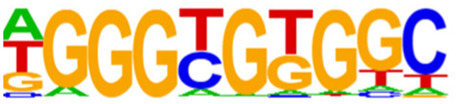 KLF5(Zf)/LoVo-KLF5-ChIP-Seq(GSE49402)/Homer	<0.001	37.88%	25.79%
18	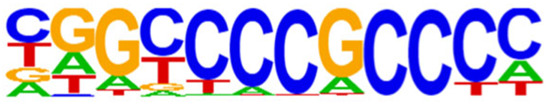 Sp2(Zf)/HEK293-Sp2.eGFP-ChIP-Seq(Encode)/Homer	<0.001	41.82%	29.46%
19	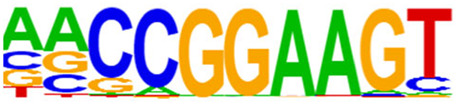 ETS(ETS)/Promoter/Homer	<0.001	9.86%	3.92%
20	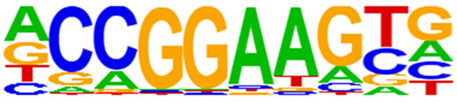 ETV4(ETS)/HepG2-ETV4-ChIP-Seq(ENCODE)/Homer	<0.001	24.29%	14.73%

## 4 Discussion

The determination of transposase-accessible chromatin involves the use of the hyperactive Tn5 transposase to cut the accessible genomic DNA and attach sequencing adaptor primers to the DNA ends to measure the openness of certain DNA regions as well as obtain important information about the open chromatin state of the entire genome of a certain cell type (Buenrostro et al., 2013; Gross and Garrard, 1988; Adey et al., 2010). This transposase preferentially inserts sequencing junctions at the unprotected regions of the DNA, thus serving as a probe to measure the genome-wide accessibility of the chromatin (Buenrostro et al., 2015). ATAC-seq technology explores how the open regions in the genome may be gene regulatory elements, such as enhancers, promoters, and TF-binding regions often enriched for TF-specific binding sites, which share similar DNA sequence patterns (motifs).

By collecting PBMC suspensions from breast cancer patients and normal controls, we identified five TFs that were highly expressed in breast cancer patients: NFY, Sp 2, GFY, NRF, and ELK 1. Four of these TFs have already been reported in breast cancer. The nuclear transcription factor Y (NFY) is a cancer-promoting gene that enhances the value-added invasion and metastasis of breast cancer by promoting the expression of proline-rich 11 (PRR 11) (Wang et al., 2019). The Sp 2 TF regulates the biological functions in breast cancer by modulating the mitochondrially related differentially expressed genes (mrDEGs) (Yan et al., 2021). Inhibition of the NFKB (NRF) TF along with non-coding the RNA TROJAN has been shown to abolish CDK2 activity and reverse the resistances of breast cancer cells to CDK4/6 inhibitors (Jin et al., 2020). The ELK 1 TF inhibits cell proliferation in breast cancer along with the tumor suppressor small non-coding RNA 135a (miR-135a) (Ahmad et al., 2018). As a new discovery in this work, the olfactory signaling factor (GFY regulator) has not been evaluated for its role in breast cancer and may therefore be used as a prediction target for the diagnosis, treatment, or prognosis of breast cancer in the future.

The combined use of ATAC-seq and RNA microarray data reveal differences in the gene expressions and regulations between tumor and normal cells. In our experiments, we used the ATAC-seq data of human peripheral blood samples from a public database RNA chip and found nine DEGs, namely JUN, MSL2, CDC42, TRIB1, SERTAD3, RAB14, RHOB, RAB40B, and PRKDC. Eight of these genes have already been reported in breast cancer. JUN can be divided into cellular JUN (c-JUN) and viral JUN (v-JUN). c-JUN is a member of the activated protein-1 (AP-1) TF family that is stimulated by upstream signals and can be transmitted by the JUN N-terminal kinase (JNK) to regulate gene expressions at the transcriptional level, thereby inducing cancer (Vogt, 2001). c-JUN is a potential regulator that stimulates the transformation of breast cells into HR+/HER2-type breast cancers (Zhu et al., 2022). The cell division control protein 42 homolog (CDC42) is frequently upregulated by several cell surface receptors and breast cancer oncogenes, as noted by Cruz-Collazo et al. (2021); the CDC42 inhibitor inhibits infiltration and metastasis of triple-negative breast cancer cells while also inducing cell cycle arrest and apoptosis of HER2-overexpression-type breast cancer cells. It reduces tumor growth and metastasis while inhibiting the migration and invasion of HR+/HER2-type breast cancer cells (Khan et al., 2020). SERTAD3 is a pro-cancer gene located within the 19q13 amplicon that has been shown to inhibit the growth of breast cancer cells and enhance tumor sensitivity to treatment with the drug tamoxifen (Li et al., 2021a). The RAS homolog family member B (RHOB) gene acts as a tumor suppressor and is the guanosine triphosphate enzyme of the RHO family; some researchers have found that RHOB plays an important role in inhibiting breast cancer invasion and metastasis (Wieland et al., 2021), and reducing RHOB expression can increase the migration and invasion capacities of triple-negative breast cancer cell lines. Restoration of the breast cancer 1 (BRCA 1) gene expressions in BRCA1-mutant triple-negative breast cancer cell lines can increase the expression of RHOB, resulting in reduced migration capacity. These results suggest that RHOB protein and BRCA1 mutations are potential therapeutic targets for breast cancer (Privat et al., 2020). RHOB alters the hormonal responses of breast cancer cells by affecting the expressions of the estrogen receptors (ERs) and progesterone receptors (PRs). We have shown that RHOB regulates the expressions of ERs and controls their protein and mRNA levels; furthermore, RHOB regulates the expressions of PRs by enhancing the recruitment of ERs and other major coregulatory factors to PR gene promoters. A major consequence of RHOB regulation is that it differentially affects the proliferation of breast cancer cell lines. It was earlier demonstrated that RHOB promotes the expressions of ERs and PRs in a manner related to cell proliferation in human breast cancer (Médale-Giamarchi et al., 2013). Some investigators found that RHOB expression was upregulated after treatment with atorvastatin, implying the potential application of RHOB as a target for tumor suppressor gene therapy in breast cancer (Ma et al., 2019). The recombination process of cellular programs in malignant cells is a stage where the tumor is very vulnerable. The male-specific lethal 2 homolog (MSL 2) gene suppresses tumor proliferation through disruption-induced excessive chromosomal instability (CIN) (Valsecchi et al., 2021). Hence, targeting MSL may be a valuable approach to treating tumors by increasing the CINs beyond the levels tolerated by cancer cells without inducing serious side effects (Monserrat et al., 2021) in normal tissues. For example, in hepatocellular carcinoma (HCC), MSL 2 overexpression has been found to partially block the inhibitory effects of the miRNA-296-3p tumor suppressor gene mode for proliferation and migration of the HCC cells, which could be used as a target for HCC therapy (Li et al., 2021b). It was also shown that MSL 2 plays a role in maintaining a normal histone modification profile that contributes to the repair of DNA damage (Lai et al., 2013). However, the role of MSL 2 in breast cancer has not been reported in other studies; hence, it may be used as a future therapeutic target in breast cancer. Tribbles pseudokinase 1 (TRIB1) is a pro-cancer gene involved in cancer initiation and progression, which could be used as a biomarker for the diagnosis and prognosis of diseases. Studies have shown that both overexpression and knockdown of TRIB1 in myeloid cells promote the growth of breast tumors in mice; myeloid TRIB1 is a negative regulator of the antitumor cytokine IL-15. Increased expression of myeloid TRIB2 reduces IL-15 levels in breast tumors, resulting in reduced numbers of T cells that are key to the antitumor immune responses. Thus, the roles of TRIB1 in chemotherapeutic responses in human breast cancer are critical and provide mechanistic insights into the importance of controlling myeloid TRIB 1 expression in breast cancer development (Kim et al., 2022). TRIB1 can also be developed as a biomarker for direct targeted therapy and predicting treatment responses (McMillan et al., 2021). RAB14 inhibition mediated by miR-320a suppresses cell proliferation, migration, and invasion in breast cancers. It has also been shown that RAB14 is a miR-320a target in breast cancer; thus, silencing RAB14 inhibits proliferation, migration, and invasion of breast cancer cell lines (Yu et al., 2016). However, RAB14 actively interacts with Nischarin by regulating the production of exosomes in breast cancer cells, subsequently affecting tumor cell adhesion, cell migration, tumor growth, and metastasis (Maziveyi et al., 2019). RAB40B is also a member of the RAS family of oncogenes and plays an important role in breast cancer cell formation, invasion, and metastasis (Jacob et al., 2013). DNA-dependent protein kinase (PRKDC) has been shown to modulate tumor sensitivity to chemotherapy and is a potential prognostic and predictive indicator of the efficacy of adjuvant chemotherapy in cancer patients. Some studies have shown that PRKDC expression is significantly higher in breast cancer tissue samples; high expression of PRKDC is also associated with a higher tumor grade, positive lymph node metastasis, and chemoresistance. Furthermore, PRKDC downregulates the sensitivity of the HR+/HER2-type breast cancer cells (MCF-7 line) to chemotherapeutic agents *in vitro* and in xenograft mouse models, indicating that PRKDC is a prognostic biomarker of chemoresistance in breast cancer patients (Sun et al., 2017). High expression of PRKDC is also a prognostic marker of poor survival in breast cancer patients (Zhang et al., 2019).

## 5 Conclusion

The use of ATAC-seq technology to identify motifs has important roles in gene regulation and disease; it provides an important basis for greater understanding of the mechanisms of TF-specific binding sites as well as new ideas for the study of TFs, enhancers, and promoters and development of new drugs. The present study identifies several key genes and TFs associated with breast cancer, providing a macroscopic theoretical basis for further research in this area. Future studies should focus on the functional validation of these identified genes and their interactions with the TFs to enhance our mechanistic understanding of their roles in breast cancer progression. Such validation could offer critical insights into their potential as therapeutic targets and contribute to the development of more effective treatment strategies. The combined application of ATAC-seq and RNA-seq can provide complementary results in tumor genomics research, help researchers better understand the regulatory mechanisms and expression profile changes during the occurrence and development of tumors, and improve the understanding and treatability of tumors. However, the present study is limited by its relatively small sample size. To strengthen the clinical relevance and utility of the identified biomarkers, future studies should focus on validating these biomarkers in larger cohorts. This would not only confirm their potential as diagnostic and prognostic tools but also enhance their applicability in personalized medicine for breast cancer treatment.

The TFs and differential genes identified and discovered in this study provide a macroscopic theoretical basis for breast cancer research and can be potential targets for future breast cancer treatments.

## Data Availability

The original contributions presented in the study are included in the article/supplementary material; further inquiries can be directed to the corresponding authors.
